# Molecularly Imprinted Polypyrrole-Modified Screen-Printed Electrode for Dopamine Determination

**DOI:** 10.3390/polym16172528

**Published:** 2024-09-06

**Authors:** Daniele Merli, Alessandra Cutaia, Ines Hallulli, Alessandra Bonanni, Giancarla Alberti

**Affiliations:** Department of Chemistry, University of Pavia, Via Taramelli 12, 27100 Pavia, Italy; daniele.merli@unipv.it (D.M.); alessandra.cutaia01@universitadipavia.it (A.C.); ines.hallulli01@universitadipavia.it (I.H.); alessandra.bonanni@unipv.it (A.B.)

**Keywords:** dopamine, molecularly imprinted polymers, electropolymerized molecularly imprinted polypyrrole, polypyrrole-based sensors, screen-printed electrodes, differential pulse voltammetry

## Abstract

This paper introduces a quantitative method for dopamine determination. The method is based on a molecularly imprinted polypyrrole (e-MIP)-modified screen-printed electrode, with differential pulse voltammetry (DPV) as the chosen measurement technique. The dopamine molecules are efficiently entrapped in the polymeric film, creating recognition cavities. A comparison with bare and non-imprinted polypyrrole-modified electrodes clearly demonstrates the superior sensitivity, selectivity, and reproducibility of the e-MIP-based one; indeed, a sensitivity of 0.078 µA µM^−1^, a detection limit (LOD) of 0.8 µM, a linear range between 0.8 and 45 µM and a dynamic range of up to 350 µM are achieved. The method was successfully tested on fortified synthetic and human urine samples to underline its applicability as a screening method for biomedical tests.

## 1. Introduction

Dopamine is a crucial monoamine neurotransmitter in the mammalian central nervous system. Neurotransmitters can be classified into excitatory and inhibitory depending, respectively, on their ability to promote the creation of a nerve impulse in the receiving neuron or to inhibit the impulse; dopamine belongs to both classes and has crucial roles in different body areas [1,2,3].

It is primarily produced in the adrenal medulla and nervous system [4,5]. Its biosynthesis involves the hydrolysis of tyrosine by the tyrosine hydroxylase to 3,4-dihydroxyphenylalanine (DA), which is further decarboxylated to dopamine by aromatic l-amino acid decarboxylase. Dopamine is also a precursor of two other catecholamines, i.e., noradrenalin and adrenaline; indeed, dopamine is hydrolyzed by dopamine β-hydroxylase to noradrenalin, while phenylethanolamine N-methyltransferase converts noradrenalin to adrenaline [6].

In the central nervous system, dopamine has a well-defined function in motor control, learning and cognition; on the other end, in peripheral organs, it regulates several functions, such as blood pressure, gastrointestinal motility, sodium levels, and hormone release [7,8,9,10]. Over the past few decades, various studies have demonstrated the significant impact of dopamine on immune cell function in both the central nervous system and periphery; this has become very important since dopaminergic immunoregulation can be a possible target for drug discovery and disease monitoring [11].

Abnormal dopamine levels in the human body can lead to several diseases. For example, cardiotoxicity is a signal of a high level of dopamine, which can evolve into cardiac decompensation, tachycardia and hypertension [12]. High levels of dopamine can also reflect an overactivity of the sympathoadrenal system, which can be due to different conditions, such as fatigue, anxiety, post-traumatic stress and chronic stress, or it can be correlated to conditions such as neuroblastoma, pheochromocytoma, neuroendocrine disorders and neurodegenerative diseases [13].

On the other side, low dopamine levels can be correlated to different pathological conditions such as depression, senile dementia, Parkinson’s and Alzheimer’s diseases [14,15,16].

Due to dopamine’s several physiological and pathophysiological implications, its detection is crucial for clinical diagnosis and monitoring of pharmacologic treatment.

Various analytical methods, such as high-performance liquid chromatography (HPLC) coupled with different detectors (UV, fluorescence mass spectrometry (MS)) [17,18,19,20]; capillary electrophoresis [21,22,23,24], fluorescence spectrometry [25,26,27,28] and chemiluminescence [29,30,31,32] were proposed for dopamine detection. Despite the high sensitivity, good precision and accuracy, the main disadvantages are the high instrumentation costs and their maintenance and management; moreover, they often require long steps for sample pretreatment and cannot be used for point-of-care or in situ analysis.

Electrochemical sensing is a viable alternative, and several methods have been developed for dopamine determination. In particular, voltammetric techniques have been exploited, taking advantage of the electrochemical oxidation reaction of dopamine [1,33]. However, the relatively high oxidation potential, its adsorption on the electrode surface, which leads to the formation of polymeric film onto the electrode surface and passivation of the electrode, the co-presence in some biological samples of interfering compounds, such as ascorbic acid and uric acid that have similar oxidation potentials, have led researchers to develop dopamine sensors by appropriately modifying the electrode surfaces to avoid all these drawbacks [33].

The functionalization of the electrode with receptors, thus obtaining sensors for the target analyte, is an effective strategy for improving the selectivity and sensitivity of electrochemical methods.

It is well known that bioreceptors are very specific and allow for high-sensitivity sensors; however, they have poor stability, are typically prone to denaturation over time, and can degrade at temperatures higher than 30 °C or in non-neutral pH solutions.

An effective alternative to bioreceptors is the employment of molecularly imprinted polymers (MIPs), which are described as synthetic analogs of biological antibody-antigen systems.

MIPs are obtained by template-assisted synthesis, which involves a functional monomer interacting in solution with the target analyte, which acts as a template to obtain a complex. A cross-linking agent and a polymerization initiator are added, and heating or UV radiation drives the polymerization. After the template molecules are extracted, binding cavities are created that are complementary in dimension and shape to the target analyte [34].

Several electrochemical sensors based on MIPs were developed for a wide range of applications [35,36,37,38]. The crucial issue is integrating the polymer with the electrode; different strategies have been exploited. The simplest is the chemical polymerization of a mixture containing the template, monomer, and cross-linker, which is drop-coated or spin-coated on the electrode surface, followed by thermal or UV polymerization to form a thin film. The main drawbacks of this strategy are the scarce reproducibility of the film thickness and sometimes the need for adding a plasticizer (for example, oligourethane acrylates) to give a greater adhesion of the MIP film on the electrode surface [35,39].

The current trend of integrating the MIP with the transducer surface is the use of electropolymerization. In this case, a suitable constant potential or a potential scan is applied to a three-electrode cell in a solution containing the template and an electroactive functional monomer, leading to the formation of the polymeric film on the working electrode surface (e-MIP). This strategy has several advantages: it is a fast and in situ process, it requires small quantities of reagents, and above all, a tuning of the polymeric film thickness is achieved by changing the electrochemical parameters, such as the range of the applied potential, the scan speed or the number of scans if cyclic voltammetry is used [39,40,41,42,43].

Aniline and thiophene derivatives can be employed as electroactive monomers for e-MIP synthesis, but pyrrole is the most commonly used due to its water solubility, which allows for electropolymerization in aqueous solutions and its ease of oxidation [44].

A helpful procedure for enhancing the features of polypyrrole-based e-MIP is overoxidation, with which polypyrrole loses its conductive properties. However, alcoholic, carbonylic and carboxylic groups are formed and can establish hydrogen bonds and other electrostatic interactions with the template molecule; so, after the template removal, higher affinity cavities are present inside the polymeric networks [42,45,46]. Moreover, overoxidation allows for better control of the film thickness and low and stable background current signals [46,47,48,49].

The literature reports some e-MIP-based voltammetric sensors for dopamine detection; most of them used a conventional three-electrode cell with glassy carbon as the working electrode [50,51,52,53,54].

In the present research, screen-printed cells are employed instead because of their main advantages, such as the low cost, the rapidity of measurement and the unnecessary sample transportation and preparation to a centralized laboratory that allows for direct in-to-the-field analysis. Indeed, the aim is to develop cheap, disposable devices designed for point-of-care and in situ dopamine determination with comparable performances to previously proposed electrochemical sensors [50,51,52,53,54]. Moreover, the present e-MIP-modified screen-printed sensor differs from similar devices already developed. For example, S. Chelly et al. [55] proposed a screen-printed voltammetric cell whose working electrode was modified with gold nanoparticles. Despite the pretty good characterization, a low-sensitive technique (linear sweep voltammetry, LSV) was used; the selectivity test is unconvincing and questionable, and samples at high dopamine content without interferent molecules were analyzed. The work of M. Pavličková et al. [56] presents a working electrode of molybdenum disulfide (MoS_2_) ink screen printed onto conductive fluorine-doped tin oxide substrates for dopamine detection. The paper describes the fabrication procedure and the electrode characterization well; however, the analytical performances are limited to a calibration curve, and no selectivity test or application to real samples has been conducted. In addition, the measuring cell requires a classical reference electrode to Ag/AgCl/3M KCl and a Pt counter electrode with a potentiostat suitable only for laboratory measurements. Another interesting work [57] proposed a smartphone-based electrochemical device using a screen-printed cell whose working electrode was modified with poly(3,4-ethylenedioxythiophene), chitosan and graphene. Being a device wholly designed and developed by the authors, the work showed few analytical results, and no analysis of real samples has been reported. Although it has good potential, it remains a prototype, and the method is not immediately applicable.

Conversely, in the present work, a sensor of simple realization suitable for direct analysis of DA both in the laboratory and in situ is proposed. In detail, molecularly imprinted electrosynthesized polypyrrole is deposited on the graphite-ink working electrode of screen-printed cells by cyclic voltammetry (CV), using dopamine as the template molecule. The better experimental conditions for the e-MIP formation are defined by a design of experiments (DoE). The overoxidation is performed by chronoamperometry at +1.0 V vs. Ag/AgCl-ink pseudoreference electrode, and the template removal is carried out by CV.

Differential pulse voltammetry (DPV) in phosphate buffer at pH 7 is selected for the quantitative analysis.

Interference tests and analysis of synthetic and human urine samples are performed using both the unmodified (bare) electrode and the e-MIP-based one, demonstrating the modified electrode’s higher sensitivity and selectivity, highlighting the potential of the developed sensor as a screening tool for in-to-the-field or point-of-care dopamine detection.

## 2. Materials and Methods

### 2.1. Reagents and Instruments

All reagents were from Merk Life Science S.r.l., Milan, Italy. Pyrrole (Py, 98%) was distilled by a Hickman distillation head and kept in darkness at 4 °C. Dopamine hydrochloride (DA, C_8_H_12_ClNO_2_, MQ200, powder), lithium perchlorate (LiClO_4_, purum p.a., ≥98.0%), sodium dihydrogen phosphate (NaH_2_PO_4_, ACS reagent, ≥99.0), sodium phosphate dibasic (Na_2_HPO_4_, ACS reagent, ≥99.0%), sodium sulfate (Na_2_SO_4_, ACS reagent, ≥99.0%), magnesium sulfate heptahydrate (MgSO_4_·7 H_2_O, ACS reagent, ≥98%), sodium chloride (NaCl, ACS reagent, ≥99%), potassium chloride (KCl, ACS reagent, 99.0–100.5%), calcium chloride (CaCl_2_, ACS reagent, ≥99%), ammonium chloride (NH_4_Cl, ACS reagent, ≥99.5%), potassium oxalate monohydrate (K_2_C_2_O_4_·H_2_O, ACS reagent, 99%), urea (CH_4_N_2_O, ACS reagent, 99.0–100.5%), uric acid (C_5_H_4_N_4_O_3_, ≥99%, crystalline), sodium citrate tribasic dihydrate (Na_3_C_6_H_5_O_7_·2H_2_O, ACS reagent, ≥99.0%), creatinine (C_4_H_7_N_3_O, for biochemistry), L-ascorbic acid (C_6_H_8_O_6_, analytical standard) and potassium hexacyanoferrate(II) trihydrate (K_4_[Fe(CN)_6_]·3H_2_O, ReagentPlus^®^, ≥98.5%) were used as received.

All solutions were prepared with ultrapure water.

Screen-printed cells (Topflight Italia S.P.A., Vidigulfo, Pavia, Italy) with carbon-ink working and counter electrodes and an Ag/AgCl-ink pseudo-reference electrode were employed (see the picture in Figure S1, Supplementary Material).

EmStat4s (PalmSens BV, Houten, The Netherlands) was used for voltammetric and EIS (Electrochemical Impedance Spectroscopy) analyses.

SNE 4500M Plus SEM (SEC Co., Ltd., Gyeonggi-do, Republic of Korea) was employed to acquire Scanning Electron Microscope (SEM) images.

### 2.2. Synthetic Urine Preparation

The artificial urine was prepared according to a proposed protocol [58]. Table 1 summarizes the composition of this solution. The appropriate amount of each reagent was dissolved in 1 L of ultrapure water and stored in a fridge at 4 °C before use.

### 2.3. Urine Sample Collection and Pre-Treatment

A urine sample was collected over 8 h from a volunteer in a cleaned polyethylene buttle, acidified at pH 4 with HCl, stored at 4 °C and filtered before analysis (filter paper Whatman n. 42, 2.5 μm particle retention).

### 2.4. e-MIP and e-NIP Preparation: Optimization of the Experimental Condition by DoE

Before the e-MIP (or electropolymerized non-imprinted polypyrrole, e-NIP) electrodeposition, the screen-printed cell was rinsed with ethanol and dried with a small flow of N_2_.

The experimental conditions for the e-MIP preparation were optimized through a design of experiments (DoE); in particular, a face-centered composite design (FCCD) was adopted, considering two variables (number of scans in cyclic voltammetry and ratio template/monomer) at three levels as will be described below. For the data treatment, the software CAT (R version 3.1.2) was used [59]. Here is the optimized procedure summarized.

The e-MIP-functionalized working electrode of the cleaned screen-printed cell was electropolymerized by cyclic voltammetry (CV), scanning the potential from −0.6 V to +0.8 V during three cycles (scan speed 0.1 V s^−1^) in aqueous solution of LiClO_4_ 0.1 M, pyrrole 15 mM and dopamine 3 mM. The overoxidation of polypyrrole imprinted film was obtained by applying a constant potential of +1.2 V for 2 min in phosphate buffer solution (PBS) 0.1 M/KCl 0.1 M at pH 7. The extraction of the template was performed in the same supporting electrolyte solution by 20 scans of CV in the potential range from −1 V to +1 V (scan speed 0.1 V s^−1^).

The electrodeposited, non-imprinted polymer film (e-NIP) was analogously prepared but without adding dopamine to the polymerization solution.

Figure S2 (Supplementary Material) reports a schematic of the procedure.

### 2.5. Bare, e-MIP and e-NIP Working Electrodes Surface Characterization

Before and after the working electrode modification, the active area and the double-layer capacitance were determined.

The effective area was computed by applying the modified Randles–Sevick’s equation to CV voltammograms obtained in K_4_Fe(CN)_6_ 5 mM/KCl 0.1 M at pH 7, scanning the potential from −0.2 V to +0.6 V and at different scan rates ranging from 0.02 V s^−1^ to 0.5 V s^−1^ [60,61].

The double-layer capacitance (C) [61,62,63] was determined by CV at different scan rates, scanning the potential from +0.5 V to −0.5 V in NaCl 0.1 M solution, i.e., in a potential range in which the lowest faradic current is expected. The difference between the anodic and cathodic current registered at +0.02 V was plotted vs. the scan rate; the slope of the straight line corresponds to twice the double-layer capacitance.

Additional characterization of the working electrode surface, before and after modification, was performed by electrochemical impedance spectroscopy (EIS) in K_3_Fe(CN)_6_ 5 mM/KCl 0.1 M at pH 7 as the probe/supporting electrolyte solution. Measurements were registered in the frequency range from 0.01 Hz to 10^5^ Hz with a sinusoidal potential modulation of 0.05 V at a fixed potential of 0.2 V for the bare and 0.1 V for the modified electrodes (equilibrium potential of the redox probe).

### 2.6. Study of the Electrochemical Behavior of Dopamine at the Bare and e-MIP-Modified Electrodes

Dopamine electrochemical oxidation was characterized using cyclic voltammetry. CV voltammograms were registered in PBS 0.1 M/KCl 0.1 M solution at pH 7 with a 2.5 mM dopamine concentration, scanning the potential from −0.3 V to +0.6 V and at different scan rates (from 0.01 V∙s^−1^ to 1 V∙s^−1^).

The relations between the peak potential (*E*_p_) vs. log of the scan rate (log *v*), the log of the peak current (log *I*_p_) vs. log *ν* and the current function *I*_p_·ν^−1/2^ vs. ν [64,65] were studied. Moreover, the formal potential (*E*°′), the diffusion coefficient (*D*) and the reaction order were determined as already described [66,67].

### 2.7. Quantification of Dopamine by Differential Pulse Voltammetry (DPV)

Dopamine was quantified by DPV, preparing calibration curves in 10 mL of PBS 0.1 M/KCl 0.1 M solution at pH 7 and applying the following parameters: *E*_start_ = −0.3 V; *E*_end_ = +0.6 V; *E*_step_ = 0.01 V; *E*_pulse_ = 0.025 V; *t*_pulse_ = 0.2 s; and scan speed = 0.02 V/s.

Analytical figures of merit, such as the limit of detection (LOD), the limit of quantification (LOQ), the linear range and the dynamic range, were evaluated from the obtained calibration curves. In particular, from the slope of the calibration curve, LOD and LOQ were calculated according to the following equations:

(1) $$\mathrm{LOD}=\frac{3.3\cdot s_{y/x}}{\mathrm{slope}}$$

(2) $$\mathrm{LOQ}=\frac{10\cdot s_{y/x}}{\mathrm{slope}}$$

where *s_y/x_* is the standard deviation of *y*-residual obtained from the linear regression of the data; this value can be supposed not to be significantly different from the standard deviation of replicates of blank solutions [68,69].

Instead, the dopamine quantification in the synthetic and human urine samples was performed using the standard addition method.

## 3. Results and Discussion

### 3.1. Modification and Characterization of the Working Electrode of the Screen-Printed Cell

The film of electropolymerized imprinted polypyrrole over the working electrode surface of the screen-printed cell was obtained via cyclic voltammetry (CV) in an aqueous solution of lithium perchlorate 0.1 M containing pyrrole and dopamine. Aiming to get the optimal performing polymer, two independent variables (factors) were selected: the number of scans in CV and the ratio dopamine (template)/pyrrole (functional monomer) and a face-centered composite design (FCCD), i.e., a response surface design, [70,71] was applied. It was necessary to switch to this technique since a simple factorial design previously adopted as a screening method showed a non-linearity of the model of responses.

Response surface methodology relates a response to the levels of several input variables that affect it. The form of this relationship is generally unknown but can be approximated by a low-order equation, such as a second-order model. Central composite designs are the most common and widely used to estimate second-order response surfaces.

**Figure 1:**
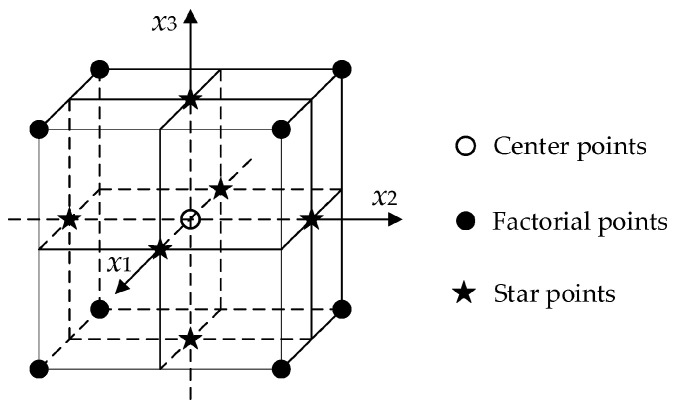
A 3-level FCCD layout. (Reproduced with permission from [72], open-access Creative Common CC licensed 4.0, MDPI).

A three-level layout for an FCCD (see Figure 1) is a cube with axial points on the face centers (star points); the center points provide information about the existence of curvature in the response surface, and the axial points allow for efficient estimation of the quadratic terms. The number of experiments required is computed by the formula: 2*^k^* + 2·*k* + n, where *k* represents the number of variables, and *n* is the number of center points. The number of experiments for 2 factors and 1 center point is 9.

Table 2 summarizes the factors selected and the corresponding levels coded as −1 (minimum level), 0 (center point) and +1 (maximum level). The slope of a three-point calibration curve obtained by plotting the current peak (*I*_p_, µA) of DPV voltammograms vs. dopamine concentration (µM) was selected as the response.

The model equation can be written as follows:

(3) $$\mathrm{slope}\;=b_{0}+b_{1}\left(n.\;\mathrm{CV}\right)+b_{2}\left(\mathrm{ratio}\right)+b_{12}\left(n.\;\mathrm{CV}\right)\left(\mathrm{ratio}\right)+b_{11}{\left(n.\;\mathrm{CV}\right)}^{2}+b_{22}{\left(\mathrm{ratio}\right)}^{2}$$

**Figure 2:**
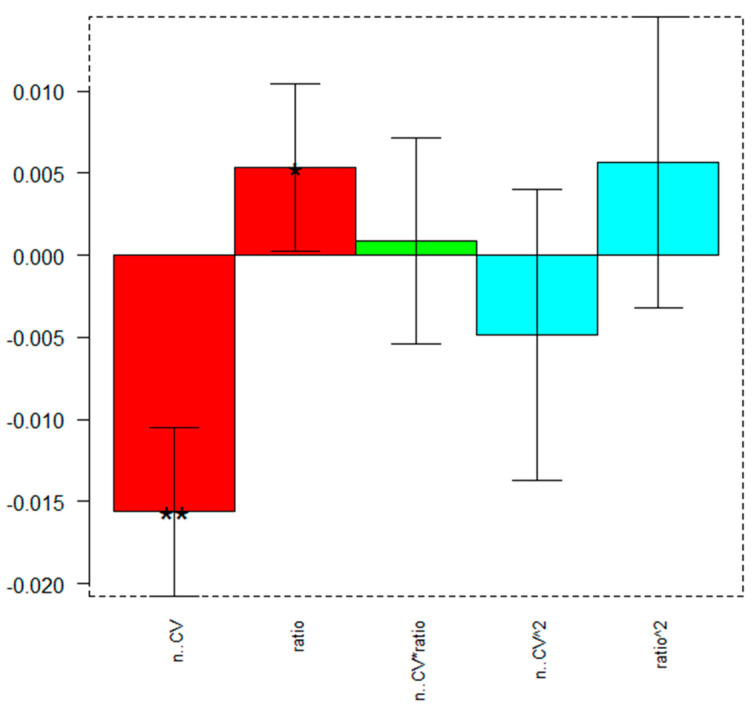
FCCD for the e-MIP film preparation: coefficients plot. Higher values (regardless of the sign) and little black stars suggest a significant influence of the respective parameter or interaction (the possible number of black stars and the relative significance are: * *p* ≤ 0.05, ** *p* ≤ 0.01).

Figure 2 reports the plot indicating the significance of the model’s coefficients and Table 3 their values.

As can be observed from the graph in Figure 2 and the values in Table 3, the significant parameters are the number of CV scans (n. CV), which must be set at the lowest value and the ratio DA/Py, which, conversely, has to be set at the highest value. The interactions and the quadratic coefficients are not significant.

**Figure 3:**
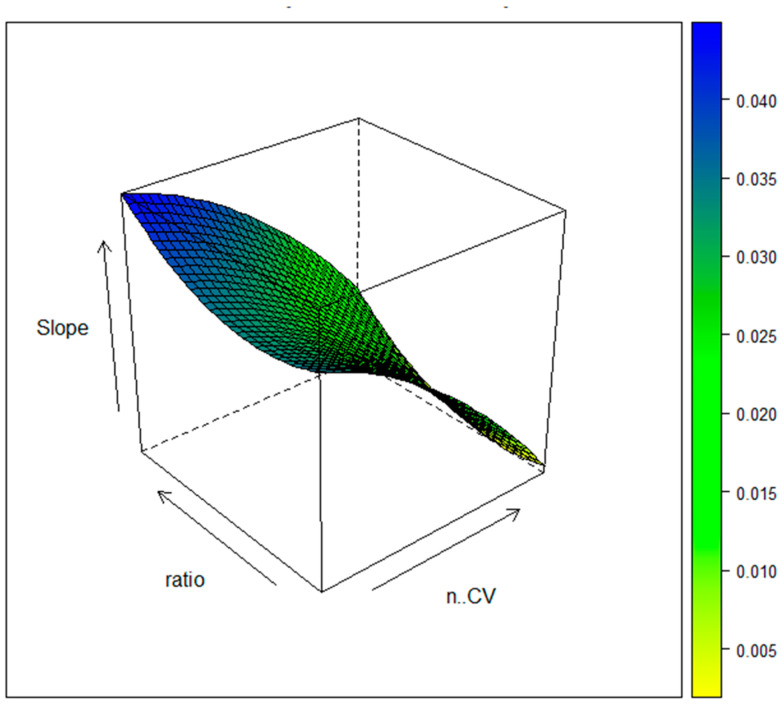
FCCD for the e-MIP film preparation: response surface plot.

The response surface plot of Figure 3 confirms the above sentence.

Three replicates at the center point were performed to validate the model. Table 4 summarizes the results (average value, standard deviation, and confidence interval (CI) at a 95% confidence level). The predicted value, i.e., the coefficient *b*_0_, is between the two ends of the CI; accordingly, the model is considered validated.

Consequently, the optimal conditions for the e-MIP film preparation are three scans in CV and a ratio DA/Py = 1/5.

### 3.2. Characterization of the Working Electrode Surface before and after Modification

The working electrode surface was characterized before and after modification with the e-MIP (or e-NIP) by determining the active area and the double-layer capacitance; moreover, the electron transfer kinetics was evaluated by electrochemical impedance spectroscopy.

Table 5 summarizes the active area and double-layer capacitance values for the three electrodes.

As can be observed, the active area decreases after modifying the working electrode surface with the polymer; moreover, the active area of the e-NIP-based electrode is lower than that of the e-MIP due to the absence of the recognition cavities, resulting in a lower active surface. This trend agrees with that previously observed for screen-printed or classical microelectrodes covered with molecularly imprinted polymers of different natures [46,61,62,64].

Concerning the double-layer capacitance for the bare electrode, the obtained value is very low if compared, for example, to that of glassy carbon electrodes, and the difference can be imputable to the structure of the carbon ink of the screen-printed electrode, with a prevalence of basal planes compared to edge plane of glassy carbon electrodes (i.e., pyrolytic graphite electrodes) that allow for faster electrochemical kinetics, as previously reported [49,63]. The double-layer capacitance increased passing from the bare to the overoxidated e-NIP and e-MIP modified electrodes, and this is due to the presence of a polymer layer, which implies an increased accumulation of electrical charges.

Cyclic voltammetry was used in this work to evaluate the capacitance, as it showed better reproducibility than other electrochemical techniques, such as EIS, for the same aim.

**Figure 4:**
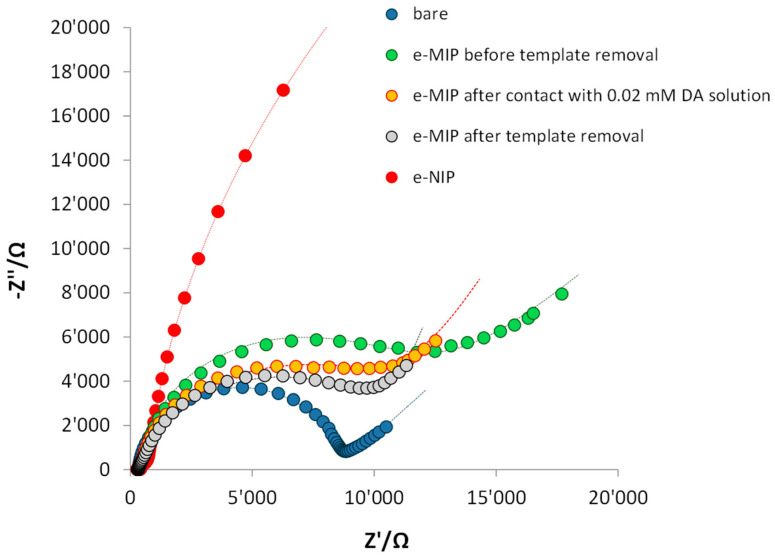
Nyquist plot of the bare electrode (blue dots), e-MIP-modified electrode before template removal (green dots), e-MIP-modified electrode after template removal (grey dots), e-MIP-modified electrode after rebinding with DA 0.02 mM (yellow dots), e-NIP-modified electrode (red dots). Measurements were performed in 5 mM K_4_Fe(CN)_6_/0.1 M KCl solution. Frequency range 100 kHz–10 mHz with a sinusoidal potential modulation of 0.05 V superimposed on a dc potential of 0.2 V for the bare and 0.1 V for the modified electrodes (equilibrium potential of the redox probe).

Conversely, EIS is employed to investigate the processes occurring at the electrolyte/electrolyte interface of the bare and modified electrodes. Figure 4 shows the Nyquist plots obtained. The bare electrode–solution interface is well modeled by a Randle’s circuit composed of two resistors, a capacitor and a Warburg element. The first resistor, representing the solution resistance, R_S_, is in series with a parallel circuit, consisting of a capacitor, C, or a constant phase element, Q, representing the electrode’s double-layer capacitance in parallel to a resistor, R_CT_, that represents the charge transfer resistance and a Warburg element, W, used to model the transfer of charge between the electrode and the redox probe species in solution and the depletion of the diffusion layer, assuming a semi-infinite linear diffusion. For the e-MIP electrode after template removal and after contact with 20 µM DA solution instead of the classical Warburg component, the equivalent circuit correlated well with the experimental data containing a Warburg short/open element accounting of the non-semi-infinite diffusion (W_S_). The equivalent circuits and the component values are reported in Table S1 (Supplementary Material).

As can be seen from the Nyquist plots, the bare electrode shows the lowest charge transfer resistance (short diameter of the semi-circle), and the diffusion contribution is evident since a straight line at 45° appears. In the modified electrodes, the charge transfer resistance increases due to the presence of a polymeric non-conducting film over the electrode surface (the polypyrrole has lost its conductive properties because it is overoxidized). Obviously, the highest R_CT_ value is registered with the e-NIP-modified electrode since, in this case, the absence of the recognition cavities prevents the probe ions from reaching the electrode surface. For similar reasons, the R_CT_ increases for the e-MIP-modified electrode switching between the free-analyte cavities of the electrode after template removal and the same electrode before extraction of DA from the recognition cavities.

SEM images (Figure S3a,b, Supplementary Material) are acquired to better display the structure and morphology of the electropolymerized imprinted or non-imprinted polypyrrole film over the electrodes. In the image of the e-MIP-modified electrode, multilayer sheets of the polymer appear, and for both e-MIP and e-NIP-modified electrodes, a lamellar structure is evident; consequently, the differences in the detection ability among the two electrodes are only attributable to the presence of the recognition cavities in the e-MIP rather than the structure and morphology of the polymeric films, as already observed [73].

### 3.3. Electrochemical Behavior of DA at the Bare and e-MIP-Modified Electrode

**Figure 5:**
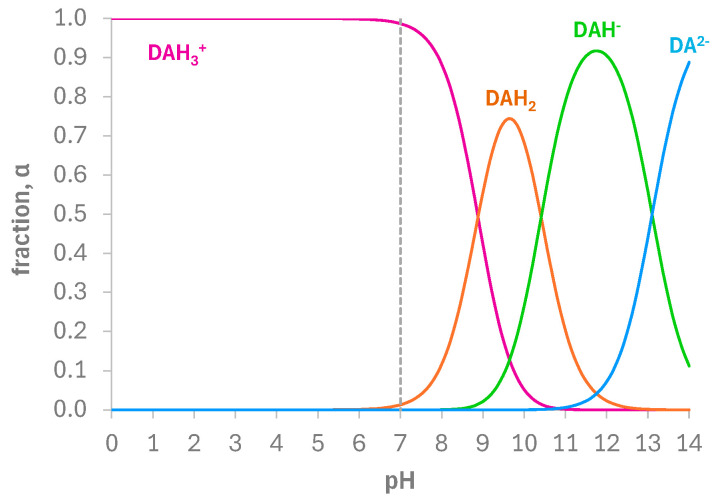
Fractions of species distribution of DA vs. pH in aqueous solution, calculated with the p*K*_a_ values reported in [74,75].

Although several electrochemical methods for DA detection have been proposed in the literature, a univocal mechanism for the electrochemical oxidation of DA has not yet been well defined and clarified, depending strictly on the operative conditions used. Furthermore, no studies have been presented using screen-printed cells. Aiming to describe this aspect and how and if the presence of the e-MIP on the working electrode surface could interfere with the electrochemical dopamine oxidation, cyclic voltammetry experiments at different scan rates in PBS 0.1 M/DA 2.5 mM at pH 7 were performed, and the redox response was examined. In these conditions, DA is in its protonated form (DAH_3_^+^), as evidenced by the fractions of species distribution graph reported in Figure 5.

**Figure 6:**
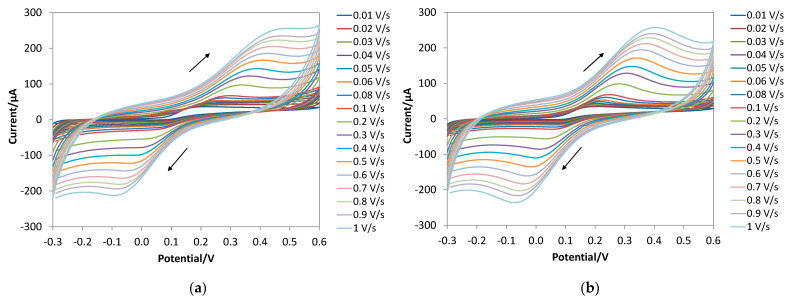
CV profile for DA (2.5 mM) in PBS 0.1 M at pH 7 at different scan rates: (**a**) bare electrode; (**b**) e-MIP-modified electrode. The arrows indicate the potential scan direction.

Figure 6 shows the CV profiles obtained with both the bare and the e-MIP-modified electrodes. Tables S2 and S3 (Supplementary Material) report the peak potential and peak current values.

As can be observed, an oxidation peak (*E*_pA_) appears at ca. 0.3 V for the bare and 0.25 V for the e-MIP-based electrodes, and a reverse reduction peak (*E*_pC_) appears at ca. 0.05 V and 0.07 V, respectively, for the unmodified and functionalized electrodes (here and in the following all potentials are referred to the Ag/AgCl pseudoreference electrode of the screen-printed cells).

It is well known that two-electron oxidation of dopamine to give dopamine o-quinone occurs with a reversibility degree dependent on the pH [76]. Since, in the present study, the difference between the anodic and cathodic peak potential is larger than ca. 30 mV and increases with the scan rate, a quasi-reversible process is suggested.

Moreover, according to previous studies [76,77], the linearity of the graph *E*_pA_ vs. *v*^0.5^ (square root of the scan rate) indicates an electrochemical reaction coupled with a chemical one, i.e., an EC mechanism (see Figures S4a and S5a, Supplementary Material).

No other peaks are present in both CV graphs, suggesting that the intramolecular cyclization of dopamine o-quinone to leucodopaminechrome (indoline-5,6-diol) does not occur. In fact, leucodopaminechrome is more easily oxidized than dopamine, producing dopaminechrome, and the reduction peak of this compound should have been observed at ca. −0.2 V [76].

A first-order chemical step following the electrochemical reaction is deduced by the slope of the plot log *I*_pA_ vs. log [DA] (Figures S4b and S5b) [78].

From the graph log *I*_pA_ vs. log *v*, a linear trend is verified with a slope near 0.5, suggesting a purely diffusive process and no adsorption phenomena occurring for both the bare and e-MIP-modified electrodes (Figures S4c and S5c).

Table 6 summarizes the electrochemical parameters characterizing the process for both bare and e-MIP-modified electrodes (the graphs used to estimate these parameters are reported in the Supplementary Materials Figures S4d,e and S5d,e).

The formal potential *E*^0^’ and diffusion coefficient *D* obtained with the bare and modified electrodes do not significantly differ from the values reported in the literature for carbon-based electrodes [77,79].

In both cases, the anodic transfer coefficient (α_A_) is similar; summing up, according to our data and previously published data, we believe that the first electron transfer is the rate-determining step. The slope of the Tafel plot also confirms this (see, for example, Figures S4f and S5f).

**Scheme 1:**
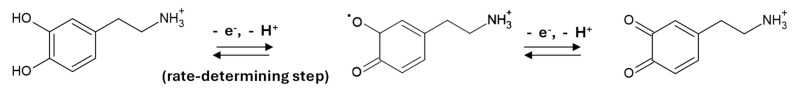
Possible pathway for the electrochemical oxidation of DA in PBS 0.1 M at pH 7.

Scheme 1 shows the hypothesized pathway for dopamine oxidation.

### 3.4. Quantification of DA by Differential Pulse Voltammetry (DPV): Calibrations and Sample Analysis

**Figure 7:**
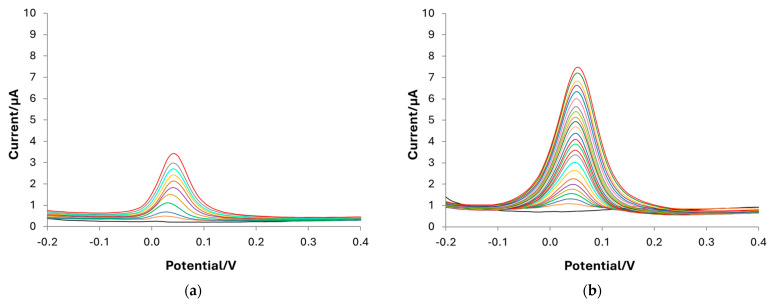

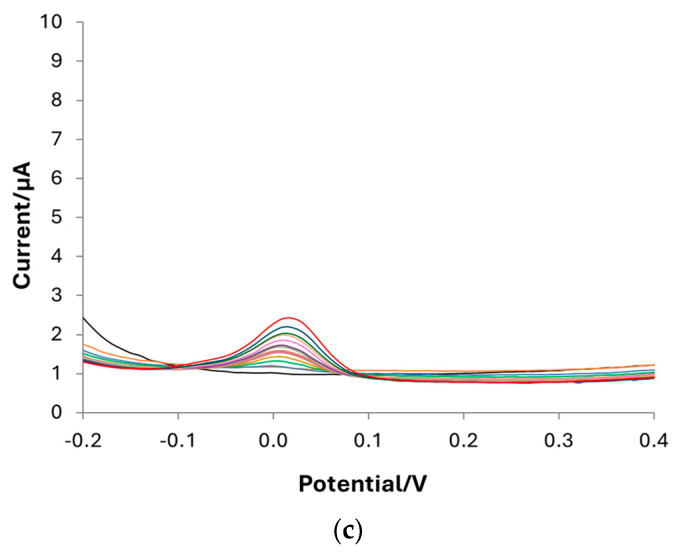
DPV voltammograms registered in PBS 0.1 M solution at pH 7 containing DA at different concentrations ranging from 0 µM to 180 µM: (**a**) bare electrode; (**b**) e-MIP-modified electrode; (**c**) e-NIP-modified electrode.

DPV is the technique selected for dopamine determination in PBS 0.1 M at pH 7. Calibration curves are carried out with bare, e-MIP, and e-NIP-modified electrodes to define the method’s figures of merit. Figure 7 shows the voltammograms registered in solutions containing DA concentrations ranging from 0 (blank) to 180 µM. As can be observed, the highest sensitivity is obtained with the e-MIP-functionalized electrode, and as expected, the performances of the e-NIP are poor due to the absence of recognition cavities so that the analyte can reach the electrode surface only through the few unspecific pores of the polymer.

**Figure 8:**
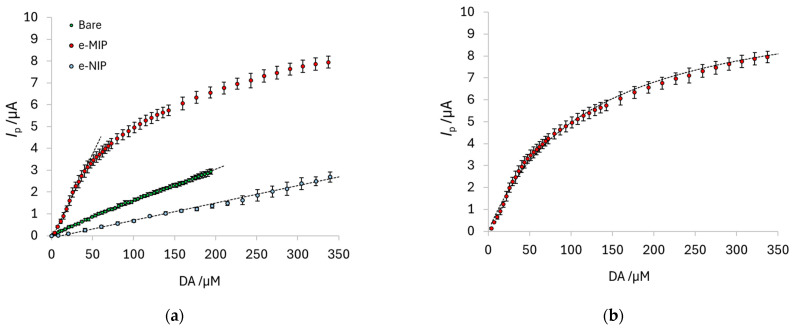
(**a**) DA calibration curves obtained with the bare, e-MIP and e-NIP-modified electrodes. Each point is the mean of the current values registered with three electrodes, and the error bars are the standard deviation. (**b**) Langmuir fitting for the calibration curve of DA with the e-MIP-modified electrode: *I*_pmax_ = 10.8 (1) µA; *K*_aff_ =8.4 (2) × 10^3^ M^−1^; R^2^ = 0.994.

Figure 8a shows the comparison among the calibration curves obtained with the different types of electrodes; each point corresponds to the mean of the current values registered with three electrodes, with the error bars representing the standard deviation. Table 7 summarizes the method’s figures of merit for each electrode type.

The linear range for the e-MIP-modified electrode covers only one order of magnitude, but the dynamic range can also be exploited to quantify DA at higher concentrations. As previously reported, the trend of the dose–response curve for MIP-modified electrodes can be well-modeled by the classical Langmuir equation [61,64]:

(4) $$I_{p}=\frac{I_{p,\max}\cdot K_{\mathrm{aff}}\cdot \left[A\right]}{1+K_{\mathrm{aff}}\cdot \left[A\right]}$$

where *I*_p,max_ is the maximum value of the current at the plateau corresponding to the saturation of the MIP’s recognition cavities, and *K*_aff_ is the affinity constant of the sites for the analyte; it is a conditional parameter since it strictly depends on the method and the experimental conditions used. Both parameters are determined by a nonlinear fitting of the data (OriginPro software version 8.5.1—Origin Lab. Corp., Northampton, MA, USA). Figure 8b shows the Langmuir fitting for the dose–response curves obtained with e-MIP-modified electrodes.

The selectivity of the e-MIP-based sensor is tested for two interferents: ascorbic acid (AA) and uric acid (UA), which are naturally present in biological fluids, such as the human urine here studied, and have an oxidation potential close to that of DA.

**Figure 9:**
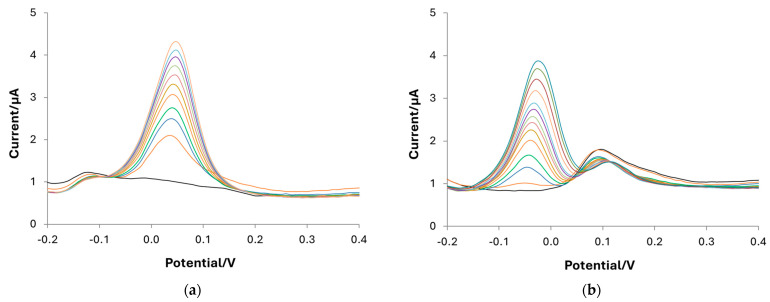
DPV voltammogram in PBS 0.1 M at pH 7 containing (**a**) 350 µM AA and (**b**) 350 µM UA with subsequent additions of DA from 0 µM to 40 µM.

Obviously, it is impossible to quantify DA in samples also containing AA and UA with the bare electrode due to the overlap of the peaks (see the voltammograms reported in Figure S6); conversely, the DA determination can be possible when using the e-MIP-modified electrode. As an example, Figure 9a,b show the DPV plots obtained in a solution containing a high concentration of the interferent (350 µM) and subsequent addition of DA from 4 µM to 40 µM. The peak separation is, in both cases, enough to quantify DA correctly. Moreover, the calibration curve for AA and UA performed with the e-MIP-based electrode showed low sensitivity and affinity constant (see Figure S7 and Table S4).

The standard addition method is considered to further confirm the possible quantification of DA in solutions containing both interferents; the recovery percentages are summarized in Table 8.

DA determination in simulated and real human urine without and with a spike of the analyte is performed to prove the developed sensor’s applicability in biological fluids analysis. Even in this case, the standard addition method is employed, and the results are also shown in Table 8.

The recovery percentage ranges from 87% to 103% for all samples, confirming the method’s suitability for DA quantification in biological fluids even in the presence of high content of interferents and without the need for sample pretreatment before analysis.

Long-time repeatability and stability tests are not performed since screen-printed electrochemical cells are disposable. They do not require particular maintenance procedures, and after modification with the e-MIP or e-NIP film, no more than two experiments (calibration or sample analysis) can be carried out with the same screen-printed cell without compromising the measurements.

## 4. Conclusions

A molecularly imprinted polypyrrole (e-MIP)-modified screen-printed sensor for dopamine (DA) determination is proposed. Conversely to previously proposed screen-printed devices, a fast, low-cost, selective method applicable in situ is here developed.

The best results were obtained after the e-MIP overoxidation, performing DPV measurements in phosphate buffer solution at pH 7 and applying optimized experimental conditions for the e-MIP film preparation.

The working electrode surface of the screen-printed cell was characterized before and after modification by CV and EIS; the results of both analyses proved the formation of a thin film of e-MIP with the selective recognition cavities for DA.

The analytical figures of merit obtained from the calibration curves demonstrated higher sensitivity and lower detection limit with the e-MIP-modified electrode compared to the non-modified (bare) one.

Tests with ascorbic acid and uric acid as interferents proved the sensor’s selectivity; indeed, the e-MIP-based electrode allows DA to be determined in the presence of both molecules even at 10 times higher concentration.

Synthetic and real human urine samples, natural and spiked with DA, were analyzed to assess the proposed method’s reliability. Recovery percentages ranging from 87% to 103% for all samples confirmed the method’s effectivity for DA quantification in biological fluids and proved its immediate applicability as a screening tool for point-of-care or in situ biomedical analysis.

## Figures and Tables

**Table 1:** The composition of synthetic urine [58].

Component	Amount (g L^−1^)
C_5_H_4_N_4_O_3_	0.25
Na_3_C_6_H_5_O_7_·2H_2_O	0.72
C_4_H_7_N_3_O	0.88
CH_4_N_2_O	1.50
Na_2_SO_4_	1.70
KCl	2.30
NaCl	1.75
CaCl_2_	0.18
NH_4_Cl	1.30
K_2_C_2_O_4_·H_2_O	0.04
MgSO_4_·7H_2_O	1.08
NaH_2_PO_4_·2H_2_O	0.29
Na_2_HPO_4_·2H_2_O	0.83

**Table 2:** Factors and corresponding levels for the FCCD to optimize the e-MIP film over the working electrode of the screen-printed cell. The label used in the following graphs (Figure 2 and Figure 3) is reported in parenthesis.

Factor	−1	0	+1
n. CV scans (n. CV)	3	5	7
DA/Py (ratio)	1/15	1/10	1/5

**Table 3:** FCCD for the e-MIP film preparation: coefficients values and their significance (the possible number of black stars and the relative significance are: * *p* ≤ 0.05, ** *p* ≤ 0.01). The number in parenthesis is the standard deviation on the last digit.

Coefficient	Value	Significance
*b*_0_	0.024(3)	
*b*_1_	−0.016(2)	**
*b*_2_	0.005(2)	*
*b*_12_	0.001(2)	
*b*_11_	−0.005(3)	
*b*_22_	0.006(3)	

**Table 4:** FCCD for the e-MIP film preparation: model validation by three replicates at the center point.

	Slope (µA µM^−1^)
Average	0.028
Standard deviation	0.005
Upper bound CI	0.037
Lower bound CI	0.019
Predicted response (*b*_0_)	0.024

**Table 5:** Active area and double-layer capacitance values for the screen-printed cells with the working electrode unmodified (bare) and modified with e-MIP and e-NIP. The active area was calculated by Randles–Sevick’s equation from CV data obtained in K_3_Fe(CN)_6_ 5 mM/KCl 0.1 M at pH 7.5 as the probe/electrolyte solution. Potential scan from −0.2 V to +0.6 V, scan rate from 0.02 V s^−1^ to 0.5 V s^−1^. The double-layer capacitance was derived from CV in NaCl 0.1 M. Potential scan from −0.5 V to +0.5 V, scan rate from 0.02 V s^−1^ to 0.5 V s^−1^. The number in parenthesis is the standard deviation on the last digit.

Electrode	Active Area mm^2^	Double-Layer Capacitance μF
bare	4.8(1)	2.8(1)
e-MIP-modified	2.0(3)	3.4(1)
e-NIP-modified	1.7(2)	3.2(2)
geometric area (circular-shaped working electrode of the screen-printed cell ø 1.2 mm)	4.5	

**Table 6:** Electrochemical parameters obtained from CV at different scan rates for the bare and e-MIP-modified working electrodes of the screen-printed cell. The number between parentheses refers to the uncertainty on the last digit.

	Bare Electrode	e-MIP-Modified Electrode
*E*^0^′/V	0.17(2)	0.16(1)
*D*/cm^2^ s^−1^	3.9(1)·10^−6^	4.5(3)·10^−6^
α_A_	0.35(2)	0.38(2)

**Table 7:** Analytical figures of merit of the DA quantification method using the bare, e-MIP and e-NIP-modified electrodes. The number between parenthesis is the standard deviation on the last digit.

	Sensitivity (µA µM^−1^)	Linear Range (µM)	Dynamic Range (µM)	LOD (µM)	LOQ (µM)
Bare electrode	0.0148(1)	10–200	-	10	30
e-MIP-modified electrode	0.078(2)	0.8–45	0.8–350	0.8	2
e-NIP-modified electrode	0.0080(1)	30–350	-	30	92

**Table 8:** Recovery test in simulated and real samples. Three replicates for each sample using three different e-MIP-modified electrodes. The number between parentheses is the standard deviation on the last digit.

Sample	DA Content (µM)	DA Added (µM)	DA Found (µM)	Recovery (%)
PBS 0.1 M pH 7, AA 20 µM, UA 5 µM	-	8	8.4(2)	103
PBS 0.1 M pH 7, AA 20 µM, UA 20 µM	-	16	16.5(2)	102
Simulated urine (1)	-	2	1.73(2)	87
Simulated urine (2)	-	4	3.6(1)	94
Simulated urine (3)	-	8	7.5(8)	93
Real human urine		-	3.31(2)	
Spiked real human urine (1)	3.31(2)	2	5.28(1)	99
Spiked real human urine (2)	3.31(2)	4	7.27(1)	100

## Data Availability

Data are contained within the article.

## Supplementary Materials

The following supporting information can be downloaded at: https://www.mdpi.com/article/10.3390/polym16172528/s1, Figure S1: Picture of the screen-printed electrochemical cell; Figure S2: Scheme of the preparation of the e-MIP-modified electrode; Figure S3: SEM images of (a) e-MIP-modified electrode and (b) e-NIP-modified electrode; Figure S4: Electrochemical behavior of DA at the bare electrode; Figure S5: Electrochemical behavior of DA at the e-MIP-modified electrode; Figure S6: DPV voltammogram in PBS 0.1 M at pH 7 containing 40 µM DA (pink line), 40 µM DA, 40 µM AA and 40 µM UA (blue line), 40 µM DA, 200 µM AA and 40 µM UA (green line); Figure S7: Langmuir fitting for the calibration curves of DA (red points), UA (yellow points) and AA (light blue points) with the e-MIP-modified electrode. The fitting parameters are reported in Table S4; Table S1: Randles equivalent circuits and component values used for the curve-fitting of the EIS Nyquist plots of Figure 4; Table S2: Peak potential and peak current values of the CV voltammograms registered at different scan speeds in PBS 0.1 M at pH 7 and DA 2.5 mM with the bare electrode (see Figure 6a); Table S3: Peak potential and peak current values of the CV voltammograms registered at different scan speeds in PBS 0.1 M at pH 7 and DA 2.5 mM with the e-MIP-modified electrode (see Figure 6b); Table S4: Fitting parameters obtained by applying the Langmuir equation to the calibration curves reported in Figure S7.
