# Supplementary material for: Molecularly Imprinted Polypyrrole-Modified Screen-Printed Electrode for Dopamine Determination
Source: Polymers (Basel). 2024 Sep 6;16(17):2528. doi: 10.3390/polym16172528 (PMC11397747; doi:10.3390/polym16172528)
Supplement: Supplementary file 1 [file polymers-16-02528-s001.zip › polymers-3164508-supplementary.pdf]

Supplementary material

Molecularly Imprinted Polypyrrole-Modified Screen-Printed Electrode for Dopamine Determination

Daniele Merli, Alessandra Cutaia, Ines Hallulli, Alessandra Bonanni and Giancarla Alberti

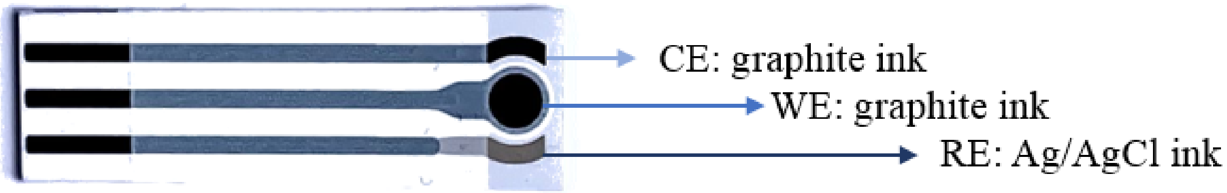

Figure S1. Picture of the screen-printed electrochemical cell

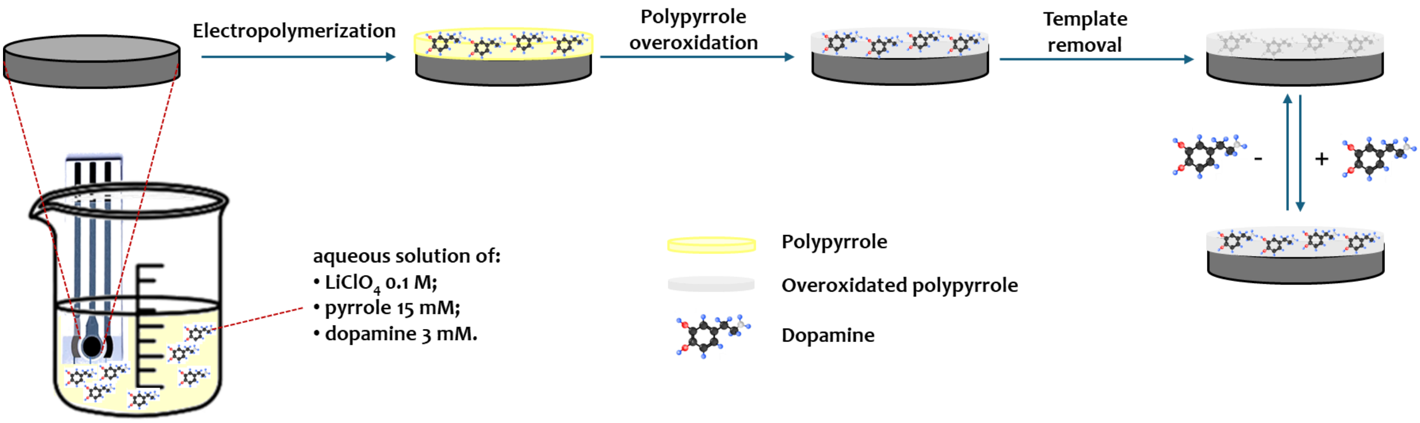

Figure S2. Scheme of the preparation of the e-MIP-modified electrode

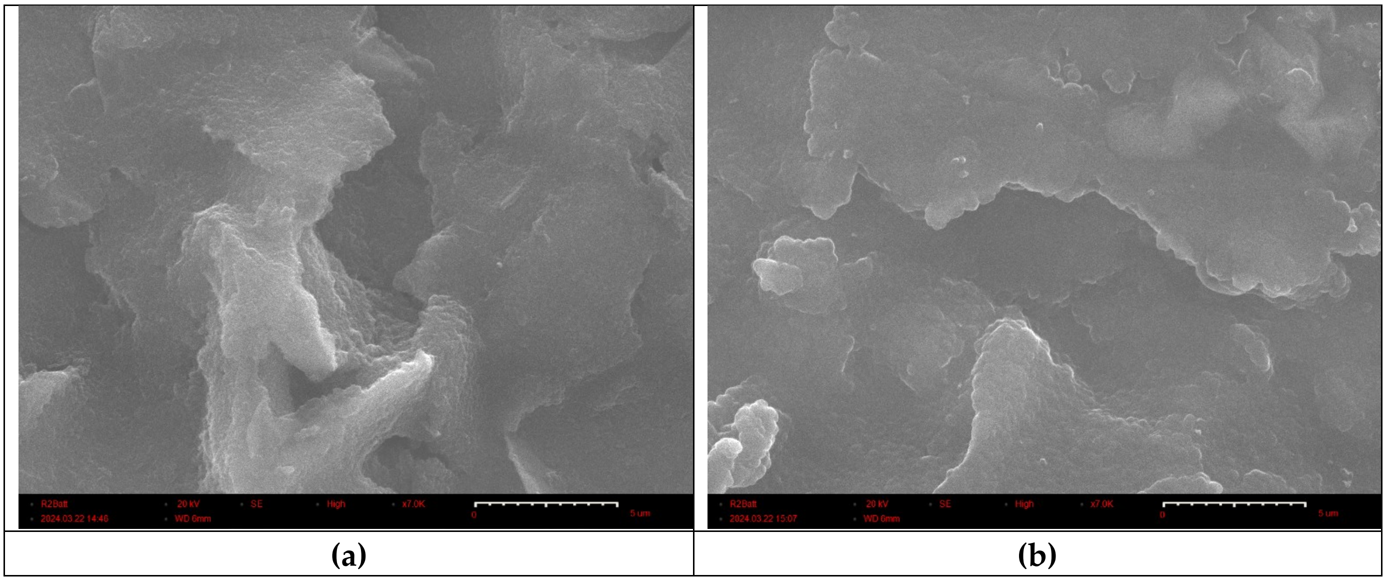

(a)	(b)

Figure S3. SEM images of (a) e-MIP-modified electrode and (b) e-NIP-modified electrode

Table S1. Randles equivalent circuits and component values used for the curve-fitting of the EIS Nyquist plots of Figure 4.

**Bare electrode**		**e-MIP electrode after template removal**		**e-MIP electrode after equilibration with DA 20 μM**		**e-MIP electrode before template removal**		**e-NIP electrode**	
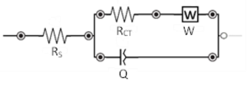		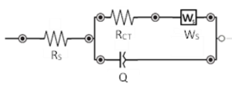		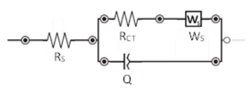		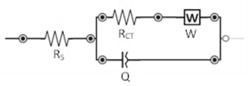		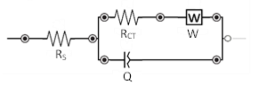	
**R_S** /Ω	322.2	**R_S** /Ω	320	**R_S** /Ω	297	**R_S** /Ω	500	**R_S** /Ω	425
**R_{CT}** / Ω	8211	**R_{CT}** / Ω	$1.1\ 10^4$	**R_{CT}** / Ω	$1.2\ 10^4$	**R_{CT}** / Ω	$1.0\ 10^4$	**R_{CT}** / Ω	$6.3\ 10^4$
**W** /σ	900	**W_S** /σ<br>/√s<br>/ ϕ	603<br>0.87<br>0.82	**W_S** /σ<br>/√s<br>/ ϕ	150<br>0.004<br>0.7	**W** /σ	2068	**W** /σ	4500
**Q** / F	$1.3\ 10^{-6}$	**Q** / F	$6.0\ 10^{-5}$	**Q** / F	$6.1\ 10^{-5}$	**Q** / F	$3.5\ 10^{-5}$	**Q** / F	$8.2\ 10^{-5}$
**n** / ϕ	0.93	**n** / ϕ	0.81	**n** / ϕ	0.82	**n** / ϕ	1	**n** / ϕ	1

Table S2. Peak potential and peak current values of the CV voltammograms registered at different scan speeds in PBS 0.1 M at pH 7 and DA 2.5 mM with the bare electrode (see Figure 6a)

**Scan rate /Vs^{-1}**	**Ep_A /V**	**Ep_C /V**	**Ip_A /μA**	**$\lvert Ip_C \rvert$ /μA**
0.01	0.19	0.08	21.09	9.25
0.02	0.21	0.08	24.73	9.22
0.03	0.22	0.08	25.38	10.78
0.04	0.23	0.07	26.94	12.12
0.05	0.23	0.07	30.65	13.76
0.06	0.25	0.06	32.42	15.05
0.08	0.27	0.06	38.34	18.10
0.1	0.29	0.05	40.61	21.47
0.2	0.32	0.03	60.39	38.32
0.3	0.35	0.01	68.50	54.53
0.4	0.37	-0.01	76.81	68.19
0.5	0.39	-0.02	88.00	81.91
0.6	0.40	-0.03	95.73	93.63
0.7	0.42	-0.05	102.5	105.7
0.8	0.43	-0.06	110.1	115.3
0.9	0.44	-0.07	116.1	124.9
1	0.46	-0.07	127.9	134.4

Table S3. Peak potential and peak current values of the CV voltammograms registered at different scan speeds in PBS 0.1 M at pH 7 and DA 2.5 mM with the e-MIP-modified electrode (see Figure 6b)

Scan rate /Vs^{-1}	Ep_A /V	Ep_C /V	Ip_A /µA	$\lvert Ip_C \rvert$ /µA
0.01	0.18	0.09	20.53	9.56
0.02	0.19	0.09	23.25	10.38
0.03	0.20	0.09	27.92	12.45
0.04	0.20	0.09	29.80	14.47
0.05	0.21	0.09	34.21	16.29
0.06	0.22	0.08	36.67	18.12
0.08	0.23	0.07	40.65	21.61
0.1	0.24	0.07	48.02	26.15
0.2	0.27	0.04	67.37	45.63
0.3	0.30	0.03	85.51	65.90
0.4	0.31	0.01	92.44	82.88
0.5	0.33	0.00	104.2	98.51
0.6	0.35	-0.02	116.6	113.1
0.7	0.36	-0.03	121.4	125.5
0.8	0.37	-0.04	128.4	136.6
0.9	0.38	-0.05	136.2	146.6
1	0.39	-0.06	144.1	156.2

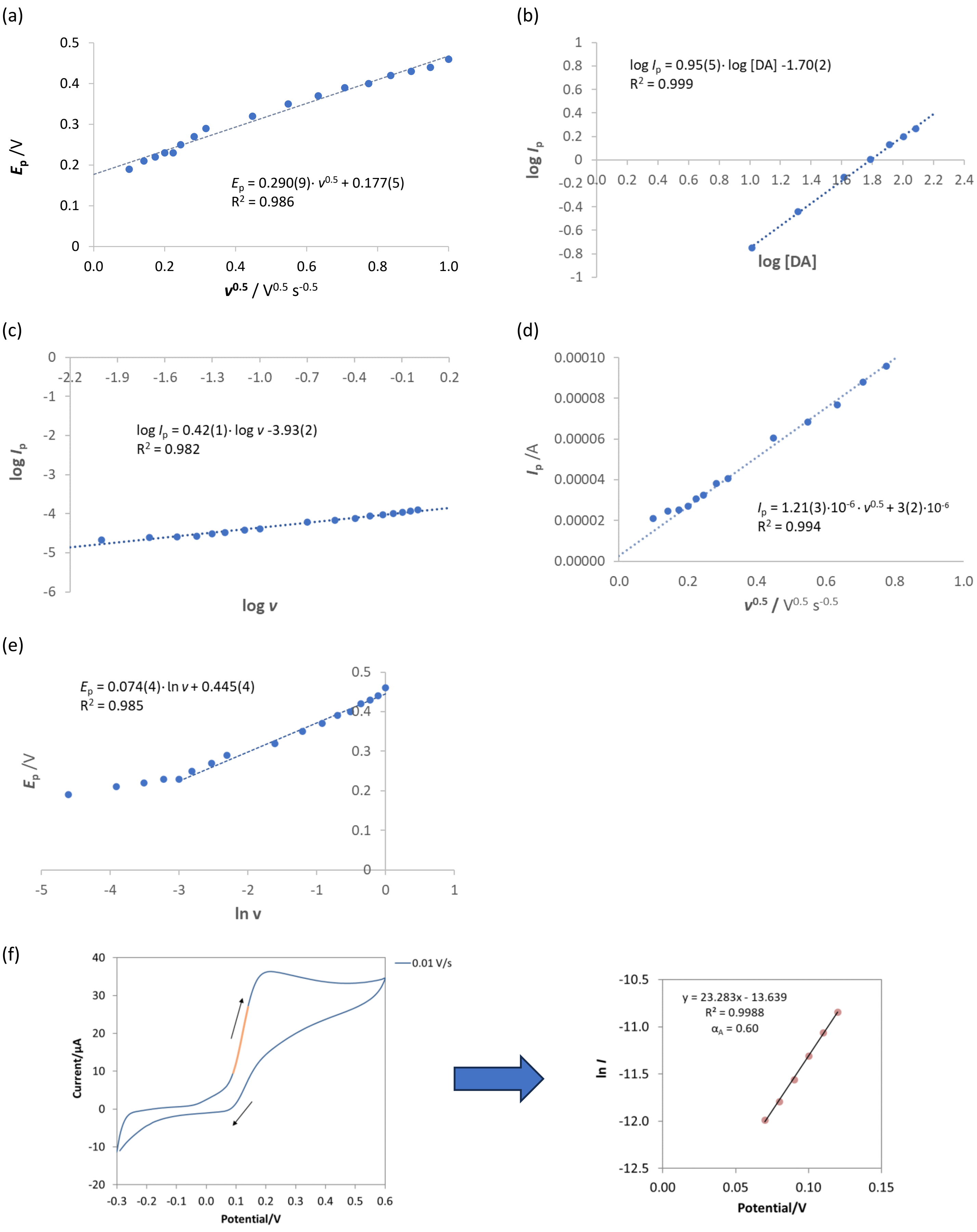

Figure S4. Electrochemical behavior of DA at the bare electrode

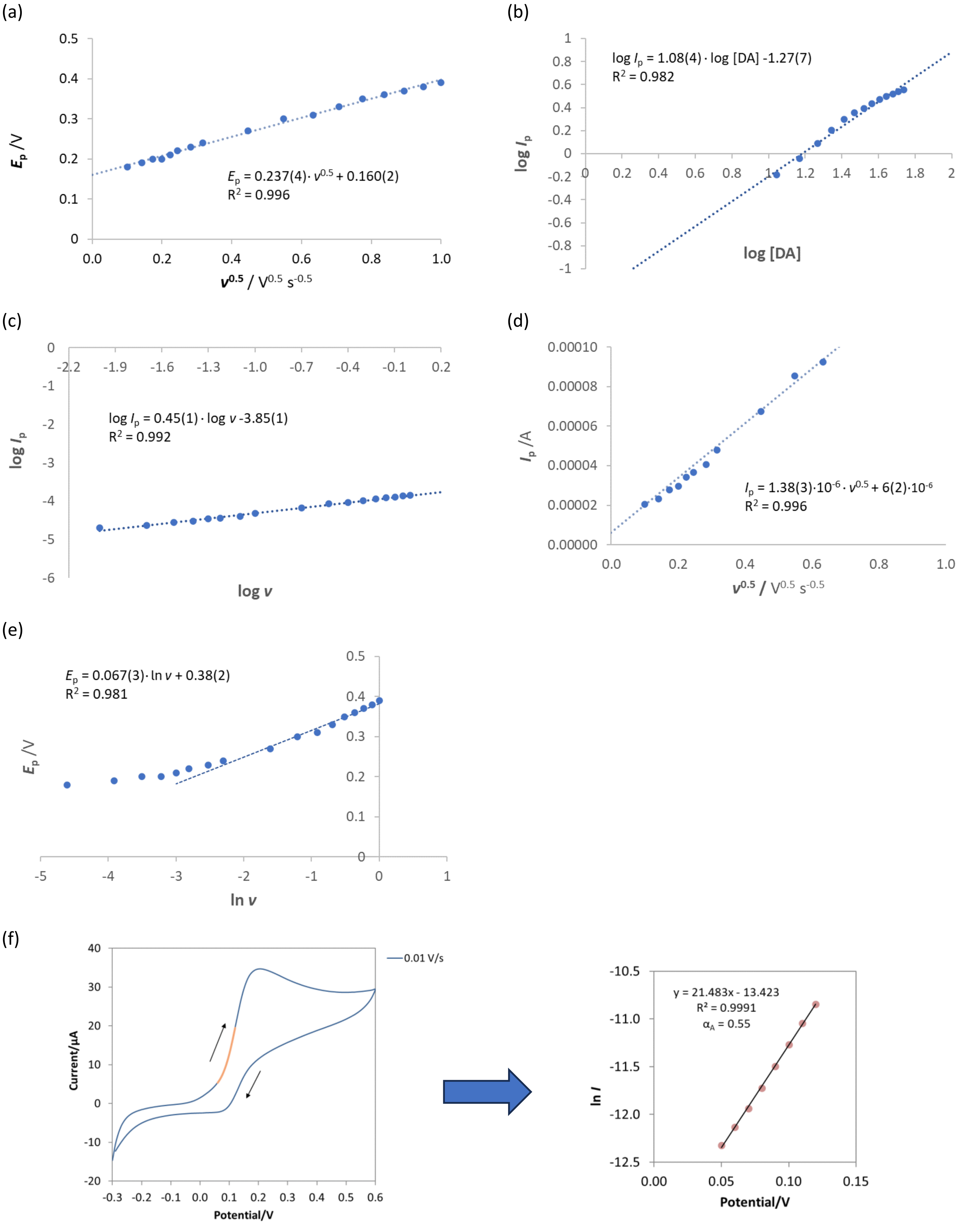

Figure S5. Electrochemical behavior of DA at the e-MIP-modified electrode

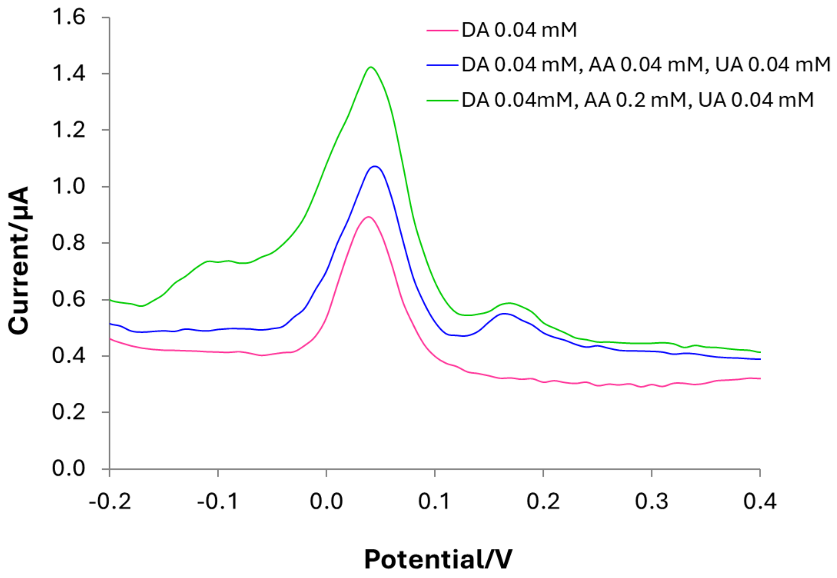

Figure S6. DPV voltammogram in PBS 0.1 M at pH 7 containing 40 µM DA (pink line), 40 µM DA, 40 µM AA and 40 µM UA (blue line), 40 µM DA, 200 µM AA and 40 µM UA (green line).

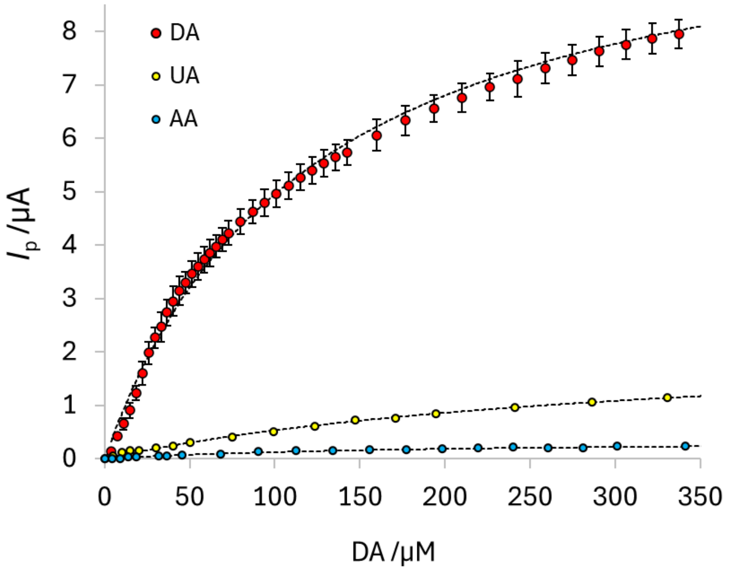

Figure S7. Langmuir fitting for the calibration curves of DA (red points), UA (yellow points) and AA (light blue points) with the e-MIP-modified electrode. The fitting parameters are reported in Table S4.

Table S4. Fitting parameters obtained by applying the Langmuir equation to the calibration curves reported in Figure S7.

analyte	$I_{p,max}$ /µA	K_{aff} /M^{-1}	R^2
DA	10.8(1)	8.4(2)×10^3	0.994
UA	2.3(1)	3.1(3)×10^3	0.992
AA	0.36(2)	5.6(6)×10^3	0.982
